# Prenatal serum sFlt-1/PlGF ratio predicts the adverse neonatal outcomes among small-for-gestational-age fetuses in normotensive pregnant women: A prospective cohort study

**DOI:** 10.1097/MD.0000000000024681

**Published:** 2021-02-26

**Authors:** So Hyun Shim, Haeng Jun Jeon, Hye Jin Ryu, So Hyun Kim, Seung Gi Min, Min Kyu Kang, Hee Jin Park, Dong Hyun Cha

**Affiliations:** Department of Obstetrics and Gynecology, CHA Gangnam Medical Center, CHA University, 566, Nonhyeon-ro, Gangnam-gu, Seoul, Republic of Korea.

**Keywords:** biomarker, fetal growth restriction, normotensive pregnancy, placental growth factor, small for gestational age, soluble fms-like tyrosine kinase-1

## Abstract

We investigated the predictive value of the soluble fms-like tyrosine kinase-1 (sFlt-1)-to-placental growth factor (PlGF) ratio for poor neonatal outcomes of SGA neonates in the absence of preeclampsia.

This prospective cohort study included 530 singleton pregnant women who attended a prenatal screening program at a single institution. The sFlt-1/PlGF values at 24 to 28^+6^ weeks and 29 to 36^+6^ weeks of gestation were analyzed and compared between control and SGA group (subdivided as with normal neonatal outcomes and with poor neonatal outcomes).

After 22 preeclampsia cases were excluded, 47 SGA neonates and 461 control neonates were included. In the SGA group, 17 neonates had adverse neonatal outcomes (36.1%, 17/47). The mean (±D) sFlt-1/PlGF ratio of early third trimester was significantly higher in SGA with averse neonatal outcome group than in the control group (14.42 ± 23.8 vs 109.12 3.96, *P* = .041) and the ratio retained an independent and significant association with SGA with adverse neonatal outcomes (odds ratio = 1.017, *P* = .01). A sFlt-1/PlGF ratio cut-off of 28.15 at 29 to 36^+6^ weeks significantly predicted adverse outcomes among SGA neonates (sensitivity = 76.9%, specificity = 88%).

In this study, sFlt-1/PlGF ratio at 29 to 36 + 6wks of SGA with adverse neonatal outcome group was significantly higher than control group. This study suggests the feasibility of the sFlt-1/PlGF ratio as helpful objective measurement for predicting the adverse SGA neonatal outcome by providing sFlt-1/PlGF cut-off value.

## 1 Introduction

Small-for-gestational-age (SGA) fetuses are defined as those having gestational weights below the tenth percentile. During the prenatal period, SGA fetuses are detected at a rate of approximately 5% to 8% of all late-phase pregnancies and usually combined clinically suspected preeclampsia (PE).^[1]^ In cases of SGA fetuses combined with PE, the fetuses are carefully evaluated for intrauterine growth restriction (IUGR) status, which can lead to fetal death due to pathologic intrauterine fetal asphyxia and should do the immediate delivery. SGA fetuses in absence of IUGR generally receive routine prenatal care with full-term delivery and have long been considered to be constitutionally small babies with a good perinatal outcome.^[2–4]^ However, as clinical study results have accumulated over recent decades, it has become clear that SGA fetuses may have poorer perinatal outcomes, from suboptimal neurodevelopment and higher postnatal cardiovascular risk to perinatal morbidity and mortality, than appropriate-for-gestational-age (AGA) newborns.^[2–9]^ Although SGA can be detected through prenatal sonogram, it is difficult to predict the poor neonatal outcome and to decide appropriate delivery time.

A well-known diagnostic tool for pathologic fetal growth restriction is the uterine artery (UtA) Doppler index.^[10–12]^ For earlier detection of fetal growth restriction, other Doppler US indices, such as increased umbilical artery pulsatility index (PI) and reduced values of middle cerebral artery (MCA) Doppler or cerebroplacental ratio (CPR), have been clinically applied.^[13]^ There is growing interest in studying combined methods for the prediction and prevention of abnormal outcomes based upon multivariable models, including US and angiogenic biomarkers, to increase sensitivity and specificity.^[14–16]^ The most widely studied angiogenic markers are placental growth factor (PlGF) and soluble fms-like tyrosine kinase-1 (sFlt-1).^[17]^ The sFlt-1/PlGF ratio is significantly higher in cases of uteroplacental insufficiency, such as PE originating from placental insufficiency.

However, clinicians must be concerned not only about fetal growth restriction combined with PE but also isolated SGA with no other abnormalities and the optimal management of such cases. There has been little research on biomarkers for poor perinatal outcomes in cases of isolated SGA in the absence of PE. A new approach and a different perspective are needed for the evaluation of isolated SGA because early recognition of SGA fetuses at increased risk of neonatal complications might enable more appropriate surveillance and, therefore, optimized management, which would subsequently reduce the risk of adverse fetal outcomes.

In the present study, we investigated the potential value of maternal serum levels of the sFlt-1/PlGF ratio for the prediction of adverse neonatal outcomes among isolated SGA fetuses in the absence of PE and investigated whether other factors can predict poor perinatal outcomes. Additionally, we reported the final analysis of clinically significant cut-off value of sFlt-1/PlGF for prediction of poor neonatal outcome in cases of isolated ultrasonic SGA fetuses.

## 2 Materials and methods

This was a prospective cohort study that included 530 singleton pregnant women who had attended a prenatal screening program at CHA Gangnam Medical Center in Seoul, Korea, between January 2011 and March 2012. Written consent was obtained from all of the participants, and the study was approved by the Institutional Review Board of CHA Gangnam Medical Center, CHA University. In our study, the control group included AGA neonates with no concerns regarding PE before or after delivery. Preeclampsia was defined as repeated systolic blood pressure measurements of ≥140 mm Hg (Korotkoff phase 1) or diastolic blood pressure measurements of ≥90 mm Hg (Korotkoff phase 5) in women who were normotensive before 20 weeks accompanied by proteinuria diagnosed as repeated ≥1 + proteinuria on dipstick urinalysis or ≥300 mg of protein in a 24-hour urine collection sample.^[18]^ Our SGA group included infants with birth weights lower than the tenth percentile of the corresponding curves for Koreans after adjustment for gestational age.^[19]^ Additionally, the SGA group was subdivided according to the neonatal outcome: SGA group with normal outcomes and SGA group with poor neonatal outcomes. The women were interviewed to obtain their obstetric data and medical history. The gestational age was assessed by embryo fetal crown-rump length in the first trimester. At a gestational age from 10 to 13 + 6 weeks, maternal serum levels of pregnancy-associated plasma protein-A (PAPP-A) were checked, and fetal nuchal translucency was measured between the gestational age of 11 and 13 + 6 weeks; subsequently, at the gestational ages of 15 to 20 + 6 weeks, 4 markers (alpha-fetoprotein (AFP), unconjugated estriol (uE3), inhibin-A, and human chorionic gonadotropin (hCG)) were measured. All of the markers were measured using a UniCel DxI 800 analyzer (Beckman Coulter Inc., Fullerton, CA, USA), and the values were transformed to multiples of the median (MoM) after adjusting for gestational age and maternal body mass index (BMI).

We sequentially analyzed 2 periods of gestational age: 24 + 0 to 28 + 6 weeks of gestation and 29^+0^ to 36^+6^ weeks of gestation.

We also measured the maternal plasma levels of the sFlt-1 and PlGF values at both early-phase gestation and late-phase gestation as additional angiogenic biomarkers. After clotting, the samples were centrifuged, and plasma was stored at − 80°C. The sFlt-1 and PlGF levels of each of the samples were measured simultaneously using the fully automated Roche Diagnostics Elecsys assay (Roche Diagnostics, Penzberg, Germany), and the sFlt-1/PlGF ratio was calculated.

In each trimester, an US scan was also performed. Fetal biparietal diameter, femur length, and abdominal and head circumferences were measured using the ATL-5000 US system (Philips Medical Systems, Andover, MA, USA). UtA Doppler ultrasonography with color-flow mapping was performed at the gestational ages of 20 weeks and 24^+6^ weeks. Both left and right UtA blood flows were examined using color Doppler imaging. The Doppler gate was placed at the proximal UtA, according to the Fetal Medicine Foundation guidelines. The PI value was measured, and the average of the measurements from the left and right UtA was used for the analysis. The Doppler measurements were performed by 3 well-trained examiners.

Adverse neonatal outcomes regarded as requiring neonatal intensive care unit (NICU) admission were attributed to subcauses including jaundice, meconium aspiration syndrome, transient tachypnea of newborn, respiratory distress syndrome, necrotizing enterocolitis, sepsis, and the requirement of ventilation. Diagnoses up to 2 months of age were included in the study. The diagnosis of jaundice was made by measuring the serum bilirubin level in the blood; total bilirubin more than 19.5 mg/dl and increases in the level of total bilirubin by more than 0.5 mg/dl per hour or 5 mg/dl per 24 hours indicated jaundice. Meconium aspiration syndrome was diagnosed when darkly colored amniotic fluid was observed along with tachypnea and hypercapnia. Transient tachypnea of newborns was diagnosed with the exclusion of respiratory distress syndrome. The diagnosis of respiratory distress syndrome was made based on the clinical findings and the chest X-ray, which showed decreased lung volume, absence of the thymus and a ground-glass-appearance pattern. Necrotizing enterocolitis was diagnosed by both radiographic findings and clinical findings, such as poor feeding, bloating, decreased activity, blood in stool and vomiting of bile. The criteria of neonatal sepsis included a total leucocyte count <5000/mm^3^, bandemia 20%, CRP >10 ng per ml, and a decrease in erythrocyte sedimentation rate (ESR) > 10 mm in 1 hour. Hypoxia was defined according to the results of the vein blood analysis, pH<7.1 at birth.

Information on pregnancy outcomes, obstetric complications and fetal outcomes, including the sex of the infant, gestational age at delivery and birth weight, were obtained after delivery.

All statistical analyses were performed using SPSS for Windows version 21.0 (SPSS Inc., Chicago, IL, USA) software. Student *t* test or the Mann–Whitney *U* test and Pearson Chi-Squared test were used to conduct univariate comparisons between groups of quantitative and qualitative data, respectively. Categorical variables are given as total numbers (n) and percentages (%) and continuous variables in means ± SDs. The comparison of maternal characteristics, angiogenic factors and UtA PI values was performed between the following groups:

1. control group vs total SGA,
2. SGA with normal outcome vs SGA with adverse neonatal outcome, and
3. control vs SGA with adverse neonatal outcome.

Values of *P* < .05 were considered statistically significant. To determine the significant factors for the prediction of SGA and SGA with adverse neonatal outcomes, logistic regression analysis with backward stepwise elimination by sequentially removing nonsignificant variables was used. Receiver operating characteristic (ROC) curves were constructed using logistic regression analysis to determine the clinically applicable cut-off value of sFlt-1/PlGF for the prediction of SGA neonates, especially those who showed adverse neonatal outcomes. The resulting areas under the ROC curves (AUCs) were compared by pairwise analysis.

## 3 Results

**Figure 1:**
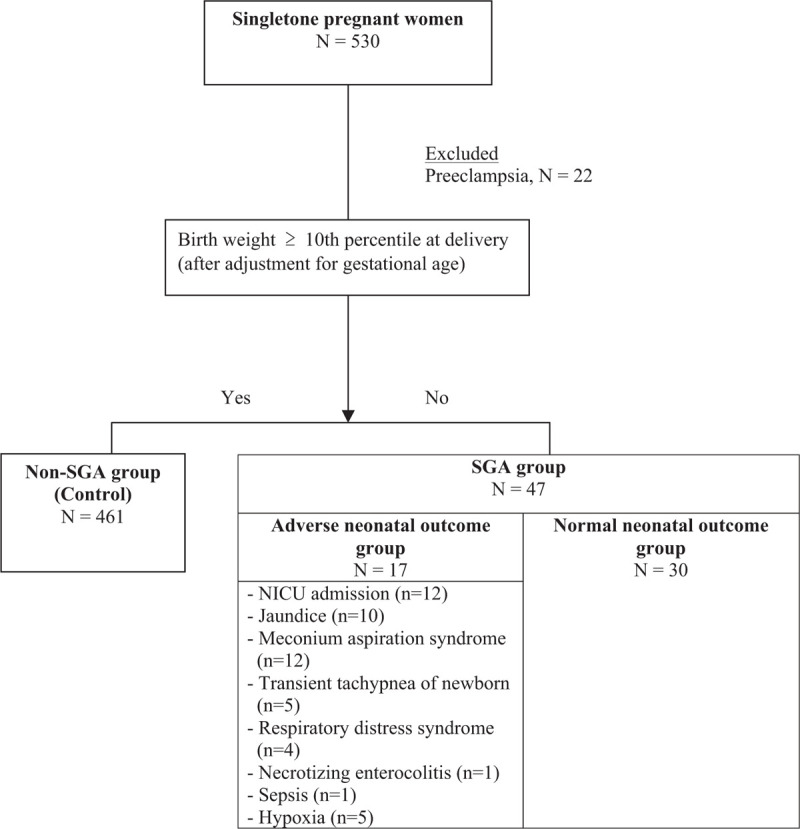
Flow diagram of participant through study.

Among the 530 study participants, 22 (4.2%) pregnant women were diagnosed with PE. After excluding these 22 PE singletons, 508 sets of pregnancy data were analyzed and grouped as 47 SGA singletons and 461 control group subjects. The neonatal outcome data are presented in Figure 1.

The comparisons of basic characteristics among the 4 groups are summarized in Table 1. Prepregnancy maternal BMI was significantly lower in the SGA and SGA with adverse neonatal outcome groups, and the percentages of primigravidarum women was high in the SGA and SGA with adverse neonatal groups, compared to the control group.

**Table 1:** Comparison of basic charateristics among the control, total SGA, SGA with normal neonatal outcome and SGA with adverse neonatal outcome groups.

	Control (N = 461)	Total SGA (N = 47)	*P*^1^	SGA with normal neonatal outcome (N = 30)	SGA with adverse neonatal outcome (N = 17)	*P*^2^	*P*^3^
Age, years (mean ± SD)	32.26 ± 4.07	31.4 ± 3.63	.169	31.23 ± 3.67	32.71 ± 3.65	.673	.583
Prepregnancy BMI, kg/m^2^ (mean ± SD)	20.21 ± 2.69	18.79 ± 1.94	<.001^∗^	18.77 ± 1.99	18.82 ± 1.91	.924	.036^∗^
Primigravida (N, %)	64.6	80.9	<.001^∗^	75.8	82.1	.312	<.001^∗^
Gestational weeks at delivery (mean ± SD)	38.43 ± 1.49	38.36 ± 1.58	.783	38.33 ± 1.64	38.41 ± 1.50	.872	.971
Gender of baby (N, %)							
Male	48.7	42.6	.421	40	47.1	.558	.65
Female	51.3	57.4		60	52.9		
Hemoglobin in early pregnancy, g/dl (mean ± SD)	12 ± 1.15	12.21 ± 0.90	.161	12.16 ± 0.81	12.28 ± 1.05	.667	.326
Gestational weeks at 1st serum test	11.45 ± 0.62	11.57 ± 0.61	.265	11.61 ± 0.58	11.5 ± 0.67	.623	.781
Gestational weeks at 2nd serum test	15.8 ± 1.9	15.79 ± 2.0	.885	15.72 ± 0.75	15.92 ± 0.49	.316	.498
Gestational weeks at sFlt-1/PlGF I measurement	26.1 ± 0.97	26.02 ± 0.95	.591	25.9 ± 1.01	26.2 ± 0.83	.25	.578
Gestational weeks at sFlt-1/PlGF II mesurement	36.27 ± 1.18	36.22 ± 1.28	.817	36.14 ± 1.356	36.38 ± 1.14	.557	.715
BP in early pregnancy, mm Hg (mean ± SD)							
Systolic	112.39 ± 15.3	110.22 ± 17.2	.202	110.97 ± 10.93	108.94 ± 9.23	.525	.206
Diastolic	66.15 ± 10.2	64.76 ± 10.3	.253	65.52 ± 7.67	63.47 ± 3.95	.314	.016^∗^

Table 2 compares the angiogenic factors and UtA Doppler results between the 4 groups. The MoM values of the PAPP-A from the 1st trimester screening test and the hCG and uE3 levels from the 2nd trimester screening test were significantly lower in the SGA groups. The sFlt-1/PlGF ratio at 29–36^+6^ weeks (late-phase gestation; ratio II), however, was significantly higher in the SGA groups. The UtA Doppler PI value of ultrasonography was significantly higher in the SGA groups.

**Table 2:** Angiogenic factors and uterine artery Doppler results of the control, total SGA, SGA with normal neonatal outcome and SGA with adverse neonatal outcome groups.

	Control (N = 461)	Total SGA (N = 47)	*P*^1^	SGA with normal neonatal outcome (N = 30)	SGA with adverse neonatal outcome (N = 17)	*P*^2^	*P*^3^
1st trimester screening test^a^							
PAPP-A, MoM (mean ± SD)	1.25 ± 0.7	1.00 ± 0.7	.036^∗^	1.10 ± 0.66	0.80 ± 0.47	.171	.024^∗^
Nuchal Translucency, MoM (mean ± SD)	0.99 ± 0.35	1.02 ± 0.33	.528	1.01 ± 0.32	1.04 ± 0.39	.762	.499
2nd trimester screening test^b^							
AFP, MoM (mean ± SD)	1.056 ± 0.49	1.085 ± 0.48	.618	1.08 ± 0.38	1.08 ± 0.38	.999	.774
hCG, MoM (mean ± SD)	1.169 ± 0.38	0.93 ± 0.4	.001^∗^	0.90 ± 0.35	1.00 ± 0.46	.457	.342
Unconjugated estriol, MoM (mean ± SD)	1.094 ± 0.29	0.99 ± 0.3	.041^∗^	0.98 ± 0.29	1.01 ± 0.35	.782	.348
Inhibin-A, MoM (mean ± SD)	1.29 ± 0.82	1.34 ± 0.9	.614	1.19 ± 0.55	1.68 ± 1.02	.054	.043^∗^
Maternal angiogenic factors at late second trimester^c^							
sFlt-1, pg/ml (mean ± SD)	1573.2 ± 958.1	1671.5 ± 1359.6	.543	1777.84 ± 1619.7	1498.72 ± 792.21	.525	.759
PlGF, pg/ml (mean ± SD)	617.1 ± 333.52	563.0 ± 365.2	.321	543.36 ± 317.24	594.99 ± 431.56	.662	.798
sFlt-1/PlGF ratio (mean ± SD)	3.74 ± 11.05	7.66 ± 27.2	.36	10.24 ± 34.47	3.45 ± 2.42	.438	.917
Maternal angiogenic factors at early third trimester^d^							
sFlt-1, pg/ml (mean ± SD)	2831.9 ± 1599.92	3237.7 ± 2025.2	.171	2820.70 ± 1266.52	4071.66 ± 2925.34	.18	.172
PlGF, pg/ml (mean ± SD)	399.8 ± 317.6	320.5 ± 321.7	.164	372.74 ± 357.56	216.78 ± 210.34	.174	.052
sFlt-1/PlGF ratio (mean ± SD)	14.42 ± 23.8	28.62 ± 38.4	.037^∗^	35.7 ± 56.4	109.12 ± 83.96	.147	.041^∗^
Doppler ultrasonography pulsatility of uterine artery^e^	0.93 ± 0.23	1.13 ± 0.5	.025^∗^	1.13 ± 0.42	1.25 ± 0.46	.397	.027^∗^

Table 3 displays the results of the Logistic regression analysis. UtA Doppler PI and the sFlt-1/PlGF ratio II had an independent and significant association with SGA and SGA with complications.

**Table 3:** Prediction of SGA and SGA with adverse neonatal outcome. Logistic regression analyses were used to estimate adjusted odds ratios (aOR) with 95% confidence intervals (CI).

		Model			
Variables	Control	SGA		SGA with adverse neonatal outcome	
		aOR (95% CI)^a^	*P*	aOR (95% CI)^b^	*P*
Doppler ultrasonography pulsatility of uterine artery	ref.	2.455 (2.345–2.570)	.027	3.934 (1.193–12.977)	.024
sFlt-1/PlGF ratio at 29 to 37^+6^ weeks	ref.	1.012 (1.011–1.013)	.025	1.017 (1.004–1.030)	.01

**Figure 2:**
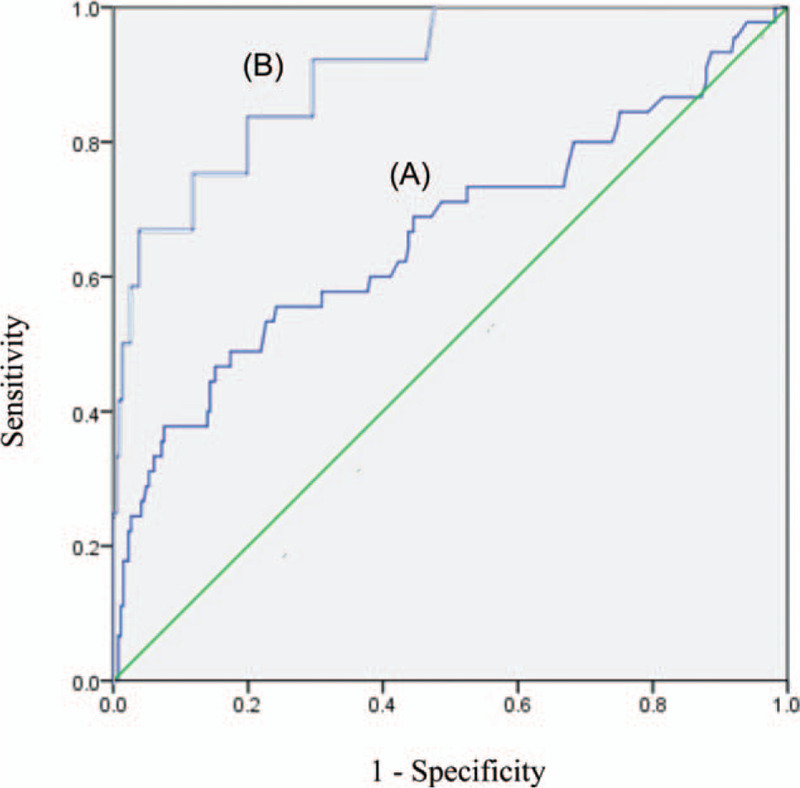
Receiver operating characteristics curves (A) for the prediction of small-for-gestational-age neonates among 508 pregnant women (461 in the control group and 47 in the small-for-gestational-age group) by the sFlt-1/PlGF ratio at 29 to 36+6 weeks of gestation, with a the cut-off value of 11.25. (B) for the prediction of small-for-gestational-age neonates with adverse neonatal outcomes among 478 pregnancy women (461 in the control group and 17 in the adverse outcomes neonatal group) by the sFlt-1/PlGF ratio at 29 to 36+6 weeks of gestation, with a cut-off value of 28.15.

Figure 2 (A) plots the ROC curves of the sFlt-1/PlGF ratio II for the prediction of SGA at birth. The best cut-off value was 11.25 with 60.0% sensitivity and 61.9% specificity (AUC area: 0.663 (95% CI, 0.564–0.762)). In Figure 2 (B), the ROC curve shows a much higher correlation between the sFlt-1/PlGF ratio II and those with SGA at birth with adverse neonatal outcomes among those subsequently admitted for NICU care. The cut-off value of the sFlt-1/PlGF ratio II was 28.15 with 76.9% sensitivity and 88% specificity (AUC area: 0.907 (95% CI, 0.829–0.985)).

## 4 Discussion

This study provides evidence that the sFlt-1/PlGF ratio measured at 29 to 36^+6^ weeks may predict SGA and SGA with adverse neonatal outcomes in addition to UtA Doppler. The sFlt-1/PlGF at 29 to 36^+6^ weeks of the SGA and SGA with adverse neonatal outcome groups was significantly higher than that of the AGA group.

Differential diagnoses between fetuses that were small due to “placental intrauterine growth restriction” and those that were “constitutionally small fetuses" have been considered a major area of interest in clinical obstetrics.^[20–24]^ Typically, ultrasonic Doppler flow indices have been used to determine the risk of adverse neonatal outcomes. UtA plays a significant role in detecting poor neonatal outcomes based on the pathophysiology of poor maternal cardiovascular function that finally causes the UtA PI value to be elevated.^[25,26]^ Our findings are in line with those of previous studies, as UtA PI was increased both in the SGA and SGA with adverse neonatal outcome groups. As another Doppler marker to predict adverse neonatal outcomes, some studies have reported that abnormal cerebral Doppler impedance is associated with poorer perinatal outcomes and neurobehavior in late-onset SGA fetuses.^[10,13,27]^ In particular, the CPR, which combines MCA-PI and UA-PI, has been reported to correlate well with adverse outcomes.^[28]^

Impaired placentation and/or placental dysfunction reflected by reduced PlGF and increased sFlt-1, in addition to such Doppler indices, is associated with the subsequent development of PE and the birth of SGA neonates. Many results have shown that imbalances in angiogenic and antiangiogenic factors, as measured in maternal blood, are detectable prior to clinical diagnosis and that such measurements have high prognostic value.^[29–35]^ The value of angiogenic biomarkers in the prediction and characterization of early-onset PE and FGR has been demonstrated in a large number of studies; already, in fact, a diagnostic serum kit based on the sFlt/PlGF ratio is being used in outpatient clinics. Gestation-age-specific sFlt/PlGF ratio cut-offs of >85 (20 + 0 to 33 + 6 weeks) and >110 (34 + 0 weeks to delivery) have been shown to be highly suggestive of PE. In the PROGNOSIS study, a single sFlt-1/PlGF ratio cut-off (<38) was validated as accurately and reliably ruling out PE within 1 week (negative predictive value >96%) and confirming PE (> = 38) within 4 weeks (positive predictive value >25%). As shown in the ASIA PROGNOSIS data, the sFlt-1/PlGF ratio is clearly meaningful for short-term prediction of PE in the suspected PE group. And even in the absence of PE, sFlt-1/PlGF ratio was associated with fetal adverse outcome. One study reported that among patients suspected of having SGA fetuses, abnormal maternal plasma concentrations of angiogenic/antiangiogenic factors were 5 to 9 and 8 to 9 times more likely to lead to PE or preterm delivery.^[36]^ Ignacio et al reported the following median values of sFlt-1/PlGF: control group 11.0, fetal growth restriction group 116.8, PE 66.5, and PE combined with fetal growth restriction group 165.4.^[37]^ In our study, we analyzed general population, not in the suspected PE group. In predicting SGA with poor neonatal outcome, the sFlt-/1PlGF ratio value showed meaningful results, lower than PE. This result shows that small increases in sFlt-1/PlGF associated with angiogenic inbalance have significance in predicting fetal weight gain and neonatal outcome.

When comparing the results with respect to gestational weeks, the sFlt-1/PlGF ratio at 24 to 28 + 6 weeks showed no significant difference in the SGA group, but there was a tendency toward increased values in the SGA group. In a recent study of the population excluding the PE group, the PlGF value investigated at a gestational age of 19 to 24 weeks (adjusted as multiples of median) was significantly lower in women with SGA infants than in those without SGA infants.^[38]^ In another paper on a population excluding those with PE, the sFlt-1/PlGR ratio was checked at a gestational age of 24 to 27 weeks, and the risk of SGA was 7.9-fold higher in women with sFlt-1/PlGF ≥90th percentile than in those with sFlt-1/PlGF <90th percentile.^[39]^ The difference from our paper is that these papers measured sFlt-1 and PlGF value at 1 time, and we measured the value serially in the late-second phase and early-third phase in the same patient. Although there is a difference in gestational weeks, these studies suggest that PlGF and sFlt-1/PlGF might be candidate biomarkers for the prediction of SGA.

There is also growing interest in the topic of sFlt-1 and PlGF in the case of isolated SGA without PE. Previous studies revealed interesting results.^[14–16]^ One case-control study reported that the combination of fetal weight, UA-PI, CPR, estriol and PlGF predicted 62% of adverse neonatal outcomes among SGA cases with a false positive rate (FPR) of 10%.^[16]^ In our study, the sFlt-1/PlGF ratio at 29 to 26 + 6 weeks was strongly predictive of adverse neonatal outcomes, with a cut-off value of 28.15 (76.9% sensitivity and 88% specificity, AUC area: 0.907). Moreover, an increased sFlt-1/PlGF ratio, with a cut-off value of 11.25, checked at the same time was shown to be a clinically predictive marker of real SGA neonates in cases of ultrasonic SGA (60.0% sensitivity and 61.9% specificity; AUC area: 0.663; 95% CI, 0.564–0.762), providing predictive value beyond the clinical and ultrasonic values. These results suggest an optimal and novel approach that utilizes the sFlt-1/PlGF ratio as a highly effective prognostic indicator of adverse outcomes in cases of isolated SGA in the absence of PE.

It must be noted that in the third trimester, unlike the first and second trimesters, SGA and placental insufficiency might not be noticeable because SGA determination is routinely made only by US biometry. Thus, in addition to US biometry measurements for the prediction of SGA, objective measurements are needed.

A limitation of our study is the small number of patients, especially regarding the subgroup analysis. Therefore, the possibility that selection bias distorted the results to some extent could not be excluded. Although our results show significant *P* values based on the logistic regression analysis, further studies with larger patient groups and more follow up measures per patient are needed to confirm these findings. There is another limitation. When making multiple comparisons using individual Student *t* test applied to each comparison, there is a chance of a type I error increases with the number of comparisons. In our study, we compared the 3 groups. In the Bonferroni correction, Student *t* test method would have a significance level (alpha set) of *P* < .017 (0.05 divided by the number of comparisons). Thus, there is a loss of significance in several comparisons, including our primary outcomes (e.g., the sFlt-1/PlGF ratio and Doppler ultrasonography of the uterine artery). The regression model strengthens our analysis, but the results need to be interpreted carefully. Another limitation is that the difference in the multivariate model shows an approximately 1 to 3% deviation in the biochemical results. It is possible that increasing the sample size would result in a loss of this significance.

This study has important clinical implications. Effective screening for the detection of adverse perinatal outcomes in cases of isolated SGA is an area of unmet clinical need. In this study, we serially evaluated not only UtA Doppler but also sFlt-1/PlGF ratio data for cases of suspected SGA, determining that the sFlt-/PlGF ratio can be an independent marker of poor neonatal outcome. Such biomarkers can be identified from a simple blood test; thus, this approach might be helpful in areas where access to Doppler examination expertise is limited. If these results are corroborated by others, the strong potential of angiogenic biomarkers for risk stratification in cases of US-detected isolated SGA and the subsequent reduction in both morbidity and healthcare costs for the management of SGA fetuses will be confirmed.

In conclusion, the sFlt-1/PlGF ratio at 29 to 36^+6^ weeks in the SGA with adverse neonatal outcomes group was significantly higher than that in the control group. These results suggest the feasibility of sFlt/PlGF ratio, based on the cut-off value of 28.15, as an objective measurement and a possible useful predictor of SGA and adverse neonatal outcomes in addition to maternal Doppler indices.

## Author contributions

**Conceptualization:** Sohyun Shim.

**Data curation:** Sohyun Shim, Haengjun Jeon, Hye Jin Ryu, So Hyun Kim, Seung Gi Min, Min Kyu Kang.

**Formal analysis:** Sohyun Shim, Seung Gi Min.

**Methodology:** Hee Jin Park, Donghyun Cha.

**Project administration:** Donghyun Cha.

**Software:** Hee Jin Park.

**Supervision:** Hee Jin Park, Donghyun Cha.

**Validation:** Donghyun Cha.

**Visualization:** Donghyun Cha.

**Writing – original draft:** Sohyun Shim.

**Writing – review & editing:** Hee Jin Park.

## Correction

The authors would like noted that Drs. Dong Hyun Cha and Hee Jin Park contributed equally. This has been updated in the footnote.
